# Role of data from cost and other economic analyses in healthcare decision-making for HIV, TB and sexual/reproductive health programmes in South Africa

**DOI:** 10.1093/heapol/czab071

**Published:** 2021-06-29

**Authors:** Joshua P Murphy, Sharon Kgowedi, Nalini Naidoo, Sarah Girdwood, Lise Jamieson, Djøra Soeteman, Stephen Resch, Gesine Meyer-Rath

**Affiliations:** Health Economics and Epidemiology Research Office (HE2RO), University of the Witwatersrand, 39 Empire Rd, Johannesburg 2193, South Africa; Health Economics and Epidemiology Research Office (HE2RO), University of the Witwatersrand, 39 Empire Rd, Johannesburg 2193, South Africa; Health Economics and Epidemiology Research Office (HE2RO), University of the Witwatersrand, 39 Empire Rd, Johannesburg 2193, South Africa; Health Economics and Epidemiology Research Office (HE2RO), University of the Witwatersrand, 39 Empire Rd, Johannesburg 2193, South Africa; Health Economics and Epidemiology Research Office (HE2RO), University of the Witwatersrand, 39 Empire Rd, Johannesburg 2193, South Africa; Center for Health Decision Science, Harvard T.H. Chan School of Public Health, 718 Huntington Ave, Boston, MA 02115, USA; Center for Health Decision Science, Harvard T.H. Chan School of Public Health, 718 Huntington Ave, Boston, MA 02115, USA; Health Economics and Epidemiology Research Office (HE2RO), University of the Witwatersrand, 39 Empire Rd, Johannesburg 2193, South Africa; Department of Global Health, School of Public Health, Boston University, 801 Massachusetts Ave, Boston, MA 02118, USA

**Keywords:** Health economics, decision-making, budgeting, South Africa, interviews, cost data

## Abstract

An increasing focus on the use of the results of cost analyses and other economic evaluations in health programme decision-making by governments, donors and technical support partners working in low- and middle-income countries is accompanied by recognition that this use is impeded by several factors, including the lack of skills, data and coordination between spheres of the government. We describe our experience generating economic evaluation data for human immunodeficiency virus, tuberculosis and sexual/reproductive health programmes in South Africa alongside the results of a series of in-depth interviews (IDIs) among decision-makers within the South African government and implementing organizations (data users) and producers of economic evaluations (data producers). We summarize results across (1) the process of implementing a new intervention; (2) barriers to the use of cost data and suggested solutions and (3) the transferability of experiences to the planned South African implementation of universal health coverage (UHC). Based on our experience and the IDIs, we suggest concrete steps towards the improvement of economic data use in the planning and the establishment of structures mandated under the transition to UHC. Our key recommendations include the following: (1) compile a publicly available and regularly updated in-country cost repository; (2) increase the availability of programmatic outcomes data at the aggregate level; (3) agree upon and implement a set of primary decision criteria for the adoption and funding of interventions; (4) combine the efforts of health economics institutions into a stringent system for health technology assessments and (5) improve the link between national and provincial planning and budgeting.

**Key messages**

- An increasing focus on the use of the results of cost analyses and other economic evaluations in health programme decision-making by governments, donors and technical support partners working in low- and middle-income countries is accompanied by recognition that this use is impeded by several factors, including the lack of skills, data and coordination between spheres of the government.
- We describe our experience generating economic evaluation data for human immunodeficiency virus, tuberculosis and sexual/reproductive health programmes in South Africa alongside the results of a series of in-depth interviews among decision-makers within the South African government and implementing organizations (data users) and producers of economic evaluations (data producers).
- Our key recommendations include the following: (1) compile a publicly available and regularly updated in-country cost repository; (2) increase the availability of programmatic outcomes data at the aggregate level; (3) agree upon and implement a set of primary decision criteria for the adoption and funding of interventions; (4) combine the efforts of health economics institutions into a stringent system for health technology assessments and (5) improve the link between national and provincial planning and budgeting.

## Introduction

Intervention cost should be an important consideration when governments make decisions about implementing a new healthcare intervention or expanding existing health services. Consideration of intervention cost should fall between considerations of effectiveness and feasibility of the intervention and the actual implementation of the intervention or service. An increasing focus on the use of the results of cost analyses and other economic evaluations in health programme decision-making by governments, donors and technical support partners working in low- and middle-income countries is accompanied by recognition that the use of these data for decision-making, budgeting, planning and benchmarking of expenditures is impeded by several factors, including wide variations in the methods and reporting of these data (Graves *et al.*, 2002; Halliday and Darba, 2003; Hughes *et al.*, 2009) and the lack of interaction between implementers and producers of cost and decision-makers (Kapiriri and Norheim, 2004). Developments such as the Consolidated Health Economic Evaluation Reporting Standards (CHEERS) checklist (Husereau *et al.*, 2013) and the Reference Case for Estimating the Costs of Global Health Services and Interventions of the Global Health Cost Consortium (Vassall *et al.*, 0000) have in the last years attempted to provide standards and promote consistency across cost analyses. Recent literature additionally emphasizes the importance of utilizing local preferences (Leech *et al.*, 2018) and local capacity to set priorities (Pitt *et al.*, 2016; Adeagbo *et al.*, 2018).

In South Africa, high-quality economic analyses are stipulated by the government to support decision-making for the private health sector, as part of recommendations for submissions to the Pricing Committee of the Directorate for Pharmaceutical Economic Evaluations of the National Department of Health (2012), which guide decisions regarding appropriate pricing in the private sector. There is no such guidance for the public sector, although the process of evidence review through the Essential Medicines List review committees does require consideration of economic aspects such as cost, cost-effectiveness and affordability, although the exact process and criteria are not publicly available (Perumal-Pillay and Suleman, 2017).

**Figure 1.**
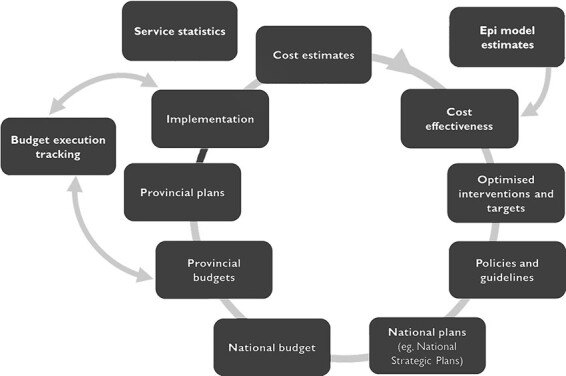
Existing framework for the HIV planning and budgeting process in South Africa

Planning and budgeting for South Africa’s public human immunodeficiency virus (HIV) and tuberculosis (TB) programmes is a case in point in that it already uses a number of economic evaluation elements summarized in the framework in Figure 1. Planning activities start with the collection of outcomes and cost data which, together with input from epidemiological and intervention models, inform cost-effectiveness and other allocative efficiency analyses (e.g. the HIV and TB Investment Case). These in turn inform government’s decisions with regard to which interventions to fund, in particular from the Conditional grant for HIV/acquired immune deficiency syndrome, TB and malaria, a central vehicle for earmarked funding under the control of the national department of health (as opposed to the remainder of the health budget, which is controlled by the nine provinces). This will then lead to budget allocations, based on considerations of both cost-effectiveness and affordability, and inform strategic or implementation plans, including intervention targets, at both the national and provincial (and increasingly district) levels. Budget execution is then tracked at all levels throughout the budget year via the continuous analysis of expenditure and performance. Finally, existing estimates (of costs, and, via timely and accessible service statistics, of outcomes) can be updated based on real-world implementation. While elements of this framework are already in place for HIV and some TB interventions, its application to other areas of healthcare provision, such as mental health services (Docrat et al., 2019), is incomplete.

To investigate how previous economic analyses of healthcare interventions have aided decision-making, budgeting and planning regarding HIV, TB and sexual and reproductive health (SRH) programmes in the public sector and how elements of the above framework can be improved, we interviewed key health decision-makers about their interactions with data from cost and other economic analyses and their experience with the process of how, when and how often these data are presented and asked them to identify facilitating as well as impeding factors for its use in health policy decision-making. We focussed our questions on decision-making for HIV, TB and SRH interventions, but also asked respondents to consider how the lessons learnt from work in these disease areas can be applied to the process of implementing the planned South African UHC scheme, National Health Insurance (NHI). NHI may merge providers and funding for the public and private health sectors over its last phase, allowing all South Africans access to healthcare free of charge at the point of use (South African Department of Health, 2017).

## Methods

We conducted a series of in-depth interviews (IDIs) among decision-makers within the government and implementing organizations working on HIV, TB and SRH programming (data users) and producers of economic evaluation and cost data (data producers). A small subset had both roles (data user-producers).

### Study design and population

We followed a top-down non-random sampling approach to identify experts as suitable respondents. Respondents were eligible if they met the following criteria: (1) employment in a relevant senior position as a manager in the government, in an academic institution/research entity or in a development organization; (2) awareness of the use of costing data and economic evaluations of health programmes in government decision-making and (3) agreement to participate and provide written informed consent for inclusion in the study. We contacted 23 potential study participants, of whom 22 agreed to participate in the interviews and enrolled in the study. Table 1 includes an overview of the type of respondents and their roles.

**Table 1.** Description of interview respondents

Type of respondent	Description of roles
Data users Data user-producers	Senior roles either in the South African government or in non-profit organizations providing programme guidance
Data producers	Academic or consultant designing and delivering cost analyses and economic evaluations from non-profit organizations or universities

### Data collection and instrumentation

IDIs were conducted over the phone, by video call, or in person, depending on the respondent’s preference. A team of at least one interviewer and one notetaker used a semi-structured interview guide to interview participants during February and March 2020 (see Table 2 for selected questions from the interview and Supplement S1 for the full interview guide). The interview guide was developed by the study team based on literature regarding data use (Measure Evaluation, 2019) and the CHEERS statement (Husereau *et al.*, 2013). We further refined the guide after a pilot test and feedback from an expert familiar with the topic. Pilot data were excluded from the final analysis.

**Table 2.** Selected questions from the interview

Topics	Selected questions
General	What are the types of programme decisions that are made in your organization? Have you ever seen outputs of economic evaluations or cost analyses? Please describe them
Use of cost data	What needs to happen to get a new intervention implemented? What are the relevant decision criteria? Who are the main stakeholders? What are the challenges and enablers to using cost data and economic evaluation? How frequently are cost data used?
Future analyses	What cost data are needed for South Africa’s NHI programme? How might HTAs be coordinated? How should interventions be prioritized? Should there be a cost-effectiveness threshold for inclusion of an intervention in the benefits package?

IDIs were carried out in English and were recorded for quality, transcription and translation purposes. Participants provided written informed consent to confirm that they had been informed about the aim of the study, the approach and the recording. Besides the recording, the interviewers took notes for each data collection event. The IDIs lasted a median of 74 minutes (range: 41–92 minutes).

### Data management and analysis

All IDIs, except for one, were audio recorded and transcribed; a single IDI was included in the analysis as ‘expanded notes’ because the respondent declined to be recorded. All transcripts were quality-checked by an interviewer who, through continuous referral to the original audio recording, verified the accuracy of the transcripts. Transcripts were then imported into NVivo 11^©^ (Doncaster, Australia), coded line-by-line and analysed using a content analysis approach (Richards, 2005).

We developed a codebook based on the literature (Husereau *et al.*, 2013; Measure Evaluation, 2019), allowing additional areas to emerge during coding. To minimize researcher bias, two coders coded the first transcript together and then coded two additional transcripts on their own and compared inter-coder or inter-rater reliability. The replies to multiple choice questions with numeric answers were recorded during the interviews and entered in a spread sheet. These data were verified during the quality check process, and frequencies were calculated. There were instances where respondents did not answer or did not want to select a response from the provided scale. These particular questions were treated as missing data, and the responses were omitted from the analysis.

We present sample characteristics of IDI respondents and our results across three thematic areas: (1) the process of implementing a new intervention or guideline in South Africa; (2) barriers to the use of cost data and data from economic evaluations, and suggested solutions; and (3) transferability of experiences to the planned South African implementation of UHC.

### Ethical considerations

Ethical approval for the study was granted from the Human Research Ethics (Medical) Committee of the authors’ institute. Data collectors were trained in qualitative interviewing techniques, Good Clinical Practice, research ethics and study procedures. Respondents were not provided any compensation for their time.

## Results

### Sample description

Of the 22 experts that were interviewed, 11 were identified as data users, 7 as both data users and producers, and 4 as data producers based on the description of their professional roles (Table 3). Our respondents had experience in a variety of programmes, primarily in HIV prevention and treatment, TB and SRH, but also in the areas of vaccines, non-communicable diseases and health promotion, and disease prevention within schools.

**Table 3.** Distribution of professional roles by type of study respondents

Type of respondent	Government	Non-profit organization	University	Total (*N* = 22)
Data users	4	7	0	11
Data user-producers	2	2	0	4
Data producers	0	3	4	7

We present some direct quotes in each section given below; a fuller collection of quotes is available in Supplement S2.

### The process of implementing a new intervention or guideline in South Africa

The most commonly described catalyst for implementing a new intervention or guideline in South Africa was the emergence of new evidence or international guidelines, particularly those issued by the World Health Organization (WHO). New evidence or guidelines initiate discussions regarding implementation or further piloting of a new intervention. A respondent shared this description, which combines both WHO guidance and South African country ownership: ‘[The WHO] provides guidance to the world, but as a country our decisions are within us, here within our branch. We have our decisions and we communicate directly with WHO’ (a003, User). In some cases, international donors were identified as the primary driver of new HIV interventions in particular, primarily from a perspective of target setting and monitoring and evaluation; one respondent mentioned that ‘PEPFAR is driving the [PrEP] process’ (a012, Producer).

Another respondent acknowledged the tension between the high-quality guidelines that the country has in place, often based on international guidance, and the lack of funds to afford their full implementation in the public sector: ‘Put together these national policies are world class, but we do not have world class amounts of money’ (a020, Producer).

Thereafter, respondents reported a complex series of trade-offs and criteria to decide which intervention should be rolled out, as well as where, to whom and how. The primary driving factor was to improve the health of all South Africans. Other factors included a substantial health innovation, which may make a case for an intervention even in the absence of substantial cost data, as in the case of the human papillomavirus vaccine.

*An intervention that can prevent cancer of the cervix. It’s like a landmark scientific development- it can prevent cancer of the cervix [* *…], and so if you don’t have the data, […] you can’t make a decision, but even if you haven’t got an exact cost effectiveness threshold, once you start getting the data, then you start getting a feel of ‘Okay, well this is how many lives are going to be saved by this intervention* (a006, User-producer).

HIV pre-exposure prophylaxis (PrEP) was another example that chronicled the many iterations of balancing priorities between available resources and policy priorities.

Table 4 includes decision factors for implementing an intervention that respondents mentioned. This table broadly mirrors how we coded and made sense of respondents’ contributions. ‘Economic’ refers to those criteria such as available funding and cost; ‘Disease specific’ is how we summarized views that discussed the epidemiology of a disease type; ‘Intervention specific’ includes factors associated with a medicine or health intervention and its corresponding strengths and weaknesses; ‘Planning/process’ is a placeholder for all those criteria involved in the conceptualization and roll-out of an intervention; and lastly, ‘Programmatic’ is a specific set of criteria used to describe resources and constraints.

**Table 4.** Decision criteria for implementing an intervention mentioned by respondents (categorized by the authors)

Decision criteria for implementation	
Economic	Planning/process
Available funding	Political will
Cost	Guidelines
Cost-effectiveness	Timelines
Cost savings	Programmatic
Disease specific	Complexity of the intervention
Burden of disease	Human resources
Need	Location
Target population	Supply chain limitations
Intervention specific	Volumes
Lives saved and other effectiveness measures (DALYs and QALYs)	Other: Equity (human rights)
Quality of evidence	
Safety	
Technological breakthroughs	

DALYs: disability-adjusted life-years.

QALYs: quality-adjusted life-years.

Next, respondents were asked to identify key stakeholders in the decision-making process. In South Africa, as in many other countries, this list is extensive, and respondents’ answers pointed to some of the challenges that may exist towards optimal cost data use due to the many stakeholders involved in the decision-making process.

*We want to roll out circumcision in schools but then we need the Department of Basic Education on board and the Department of Health who is going to provide services but then we also have the Departments of Arts and Culture and [Department of] Social Development again and to try and orchestrate those ministries in order to achieve a* *particular outcome is again challenging and because of resource constraints […] and sometimes there is [also] a lack of will* (a017 (Producer)).

### Barriers to the use of cost and economic evaluation data and suggested solutions

As reflected in the previous quote, there were tensions reported by respondents that result from sub-optimal collaboration between government structures (including the Department of Health and National Treasury). It was suggested that this particular limitation could be mitigated by building internal research capacity and health economic skills

*The department has to build its own capacity […] That’s the first thing that should happen where we have the biggest gap. […] But then there should also be a collaborative work in closer proximity between departments, either a research unit with policy development, research and policy, [or] policy units working with the health economic unit in the department, and then [health] programmes as well* (a002, User).

Other possible solutions to a lack of coordination or available skills that were mentioned by the respondents were more general and included calls to increase government’s leadership in terms of mandating economic evaluations as well as collaboration between the government and organizations with capacity to conduct these evaluations. A number of research units based at, or associated with, the authors’ institutes, the University of Cape Town and the University of KwaZulu-Natal, were mentioned.

A second, commonly identified barrier concerned the quality of and access to cost and programme outcome data. This was accompanied by the acknowledgement from respondents that the quality of HIV and TB cost and programme data was generally of higher quality, granularity and completeness compared to data in other disease areas in the public health sector, e.g. non-communicable diseases. Solutions offered by respondents included a preference for real-world (bottom-up) costing over ingredient-based costing, increased capacity building of government employees from all levels of the health system, and a continued emphasis on promoting data quality.

Lastly, with regard to the frequency with which respondents used cost data, this varied from rarely (on an annual or 5-yearly basis) to often (every day). Data producers and data user-producers reported using cost and other economic data almost every day while data users reported less frequent use, e.g. quarterly, or as infrequent as every five years for the preparation of national strategic plans. One possible solution mentioned to improve the use of cost data was to increase the frequency of mandated costing and budgeting exercises, including the frequency of such strategic plans.

### Transferability of experiences to optimize implementation of universal healthcare

With regard to how the experience with data from cost analyses and economic evaluations could be used in facilitating the roll-out of South Africa’s NHI in the coming years, the majority of respondents (21/22) indicated that the development of a network of academic institutions that can participate in the technical work involved in health technology assessments (HTAs) would be useful. There was also mention of the National Treasury’s HIV conditional grant as a helpful model that could be rolled out to other disease areas and guide aspects of NHI.

*[W]hat Treasury has done is they have worked out that the conditional grant model has been successful and they have worked nicely with [* *…] health economists that have been creating the data and the evidence for the investment cases […]. Treasury likes the investment case thing a lot. It gives them a way of controlling the National Department of Health, which I think they also like, and it gets them some way of knowing how their money has been spent […]. It is quite an interesting movement and for me it is a precursor to the NHI Benefit Package idea* (a020 (Producer)).

Some respondents (4/22) highlighted available international models that could provide valuable guidance in the design of such an HTA network, such as the Health Intervention and Technology Assessment Program in Thailand and the National Institute for Health and Care Excellence in the UK. There was, however, resistance to specifying a single-value cost-effectiveness threshold to determine whether an intervention is cost-effective and should be reimbursed. Some respondents stated that this was beyond their expertise, while others highlighted the difficulty of calculating or using a single amount, especially across interventions, and mentioned that using a threshold would not take affordability into account. Another respondent mentioned that while enough economic data might be available, the outcomes data needed for such cost-effectiveness analysis might not be of the same quality

*[F]or me it is not so much that the expenditure data is missing. We have expenditure data but what is missing is quite a lot of the outcomes data that you need to* *[…] measure these changes in health […]* (a020 (Producer)).

## Discussion

This study describes the findings of IDIs with key health decision-makers about their experiences on the use of cost and other economic data in healthcare decision-making in South Africa. The key lessons learnt from the interviews are threefold: (1) Respondents identified gaps regarding the use of cost data on both the supply and demand side. On the supply side, respondents felt there was space for improvement with regard to the quality of existing cost data, especially for interventions outside the HIV and TB area. On the demand side, respondents identified the economic literacy of data users in some segments of the government and especially at district and facility levels as sub-optimal. Moreover, respondents reported few formalized efforts to improve the use of outputs of economic analyses. Economic analyses were also hampered by the lack of regularly updated outcomes data, in particular outside of the HIV and TB programmes. (2) Another challenge was the tension between national and international priorities or guidelines, in particular in deciding on which, whether and how to implement a new healthcare intervention. This means that even in the presence of good and regularly updated data and analyses, decisions will often be based on political expediency towards international organizations or global co-funders of programmes, rather than local economic evidence. This was especially exemplified by the discussion around PrEP, a relatively costly intervention that is effective in HIV prevention, but not across the general population; (3) With regard to the needs of data users, we found that a number of decision criteria suggested by standard economic literature are currently being used in healthcare decision-making in South Africa, such as cost, cost-effectiveness, affordability and the ability to fully implement and target interventions in order to maximize their impact. In addition, we were apprised of additional decision criteria, such as programmatic constraints or political will. We also realized additional tensions between methods and formats preferred by spheres of the government, such as Treasury’s preference of the investment case format over other economic analyses. We also saw evidence of the disjoint between good clinical guidelines and considerations of affordability and/ or cost-effectiveness, as exemplified in the quote ‘Put together these national policies are world class, but we do not have world class amounts of money’. In an optimal decision framework, economic considerations would be included at the stage of deciding on policies and guidelines, incorporating the lack of ‘world class amounts of money’.

The strength of this study is that almost all of the stakeholders that were contacted agreed to participate in the interviews, consisting of stakeholders with a broad range of professional roles and both data users and producers. Moreover, the interviews were set up rapidly and conducted over a relatively short period of time in February and March 2020, allowing for a similar time frame within the policy and budget cycle. Our study also had several limitations. Our focus on high-level stakeholders meant that we did not obtain a deeper understanding of non-expert familiarity with costing and economic evaluation outputs. Additionally, our qualitative methodology with a focus on the South African context may not be directly generalizable to other countries or settings. Lastly, when inviting participation, we framed the intention of the study to be about the role of economic analyses conducted by our own research organization, in order to efficiently describe the scope. Since most questions were then phrased to include economic evaluations conducted by any other organization as well, we believe the impact of this to be small.

### Recommendations

**Figure 2.**
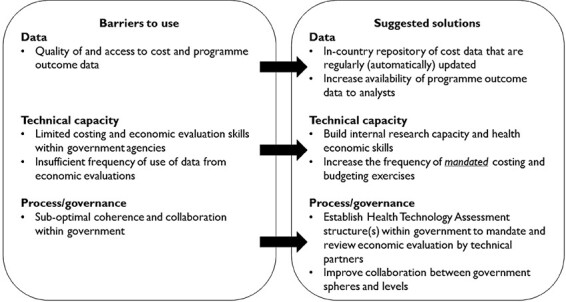
Barriers and suggested solutions to increase use of costing data and economics evaluations in South Africa

Our analysis leads to the following recommendations for improving the availability, accessibility, and use of cost and other economic data for healthcare decision-making in South Africa (see Figure 2)

1. Compile, as a first step, an in-country cost repository summarizing the results of existing cost analyses, which is publicly available and can, in a second step, be updated regularly (i.e. at least annually, ideally in an automated process informed by existing expenditure and price data, which regularly updates prices, salaries and implementation models).
2. Increase the availability and use of programmatic outcomes data at the aggregate level (district upwards) to aid the comparison of costs, expenditures and outcomes, and the calculation of costs per unit of effectiveness.
3. Agree upon and implement a set of primary decision criteria for the adoption and funding of interventions and intervention targets.
4. Better implement existing formal processes for incorporating economic information into decision-making where relevant.
5. Towards the establishment of the NHI, combine the efforts of health economics institutions into a stringent system for HTAs, mandated and commissioned by the government.
6. Increase health economics capacity within government agencies and departments to enable the commissioning of the correct types of studies and their review.
7. Improve the link between national and provincial plans and budgets and the regular updating of existing epidemiological and budget models with recent service statistics and cost estimates to improve the already established framework for HIV and TB planning (Figure 1).

## Conclusion

In conclusion, through interviews with relevant stakeholders, we were able to identify how the use and availability of intervention cost data in healthcare decision-making in South Africa can potentially be improved. We suggest concrete steps towards the improvement of economic data use in the planning of HIV, TB and SRH interventions and the establishment of structures mandating such analyses under the transition to NHI.

## Supplementary Material

czab071_Supp

## Data Availability

The data underlying this article cannot be shared publicly to protect the privacy of individuals that participated in the study. Select de-identified data (such as summary tables and a codebook) will be shared on reasonable request to the corresponding author.
