# Supplementary material for: Role of data from cost and other economic analyses in healthcare decision-making for HIV, TB and sexual/reproductive health programmes in South Africa
Source: Health Policy Plan. 2021 Jun 29;36(10):1545–51. doi: 10.1093/heapol/czab071 (PMC8597963; doi:10.1093/heapol/czab071)
Supplement: czab071_Supp [file czab071_supp.zip › Supplement 1_ Interview Guide_ v1 6feb2020.pdf]

Today's date					
d	d	m	m	y	y

Participant ID		

INTRODUCTION

Thank you for taking the time to speak with us. As mentioned in our email we would like to hear from you about the usefulness of existing economic analyses, including HE^{2}RO's work, to the national HIV, TB and sexual and reproductive health programmes.

As you know, HE^{2}RO staff and others have worked on economic analyses for the country's HIV response for the last ten years, in collaboration with the Department of Health and Treasury, and more recently also on the TB and sexual and reproductive health programmes. The ultimate purpose of these analyses is to improve these programs by enabling more informed decisions based on data regarding costs, impact, and cost-effectiveness. However, the information we and other academic organisations have provided over the years might not always have been in the most useful format, answering the most pertinent questions, or presented to the most relevant people. We are therefore conducting this analysis to inform and improve our own analytical methods in the future and also gather insight that other countries can use to improve the methods and the use of economic analyses for health decision making.

Your participation is requested to provide insight about whether HE^{2}RO's economic analyses have been useful to your organization. Your participation is very important, but is entirely voluntary. Your responses will be treated as confidential, your name, exact position and organisation will not be shared outside of the study team, and we will endeavour that any statements or comments you make cannot be linked to you as an individual. We will use the interviews to develop potential interventions to strengthen the demand for and use of the results of economic evaluations and cost analyses in decision making. Lastly, we will provide feedback to you on our findings as well as contribute to a Methods Handbook to be shared with other countries. For all of these purposes, it is important for us to receive feedback on what we and other analysts can do better, so don't hold back.

Note that for the purpose of this analysis, we define cost analyses to involve the results of all analyses that collect cost and resource use information. This information can either be collected at the level of programme implementation, from the provider or patient perspective or both, or built-up from ingredients. The results of these analyses are then often used in modelled cost-effectiveness analyses, budget impact analyses, or budgets.

Do you have any questions? May we begin?

Today's date					
d	d	m	m	y	y

Participant ID		

QUESTIONS

Part A - General

1. **Firstly, please briefly describe <u>your job title, time and role</u> in your current position and any previous roles that are relevant to this research.**

2. **What are the types of programme decisions that are made in your organization?**
   - **Probe:** For example, there may be decisions related to where to provide services, how to allocate resources or plan for new activities. How are decisions like these made in your organization?
   - **Probe:** Who is involved in the decision-making process?
   - **Probe:** What types of information do you think your organisation relies on to make decisions? (please specify)

3. **Have you ever seen outputs produced by HE^{2}RO staff, in particular outputs from economic evaluations or cost analyses?**
   If *yes*, what were they?
   *[**Interviewer guidance:** Tick "Recalled unprompted" or add under "Other"]*
   How familiar are you with the results of each?
   *[**Interviewer guidance**: Add familiarity level]*

   I will read a short list of examples of our outputs. Please let me know if you have ever heard of these studies, and if so, how familiar you are with the results.
   *[**Interviewer guidance**: Tick "Recalled prompted" and add familiarity level. If studies were already mentioned unprompted, skip these here.]*

Study	Recalled unprompted	Recalled prompted	Not very familiar	Somewhat familiar	Very familiar
HIV Investment Case					
National ART Cost Model (Other name: "National AIDS Costing Model")					
NSP costing (either 2011-2016 or 2017-2022)					
PrEP cost/ CEA					
TB Investment Case					
MDR short-course costing					
TB Xpert costing					
Breast or cervical cancer policy budgets					
Other 1:					
Other 2:					
Other 3:					

*[**Interviewer guidance:** If none of these are recalled even if prompted, skip Section C.]*

4. **Have you ever seen outputs of economic evaluations or cost analyses produced by others? If yes, what were they?**

Today's date					
d	d	m	m	y	y

Participant ID		

Part B – Use of cost data generally

5. **From your perspective, please explain the steps currently involved in healthcare decision making in South Africa. What needs to happen to get a new intervention implemented?**
   - **Probe:** For example, what needs to happen for the roll-out of PrEP, bedaquiline or the HPV vaccine?
   - **Probe:** Please include discussion of a timeline *(ie, within a budget year or the time period it takes).*

6. **Who are the main stakeholders?**

7. **What are the relevant decision criteria?**

8. **From your perspective, what elements *should* be involved in healthcare decision making in South Africa that aren't currently involved?**

9. **How is cost data currently used in these decisions?**
   - **Probe:** Explain when and where in the budget cycle cost data is of importance.
   - **Probe:** What are the elements enabling the use of cost data? organisational structures, policies, skills, data streams, funders, timeliness, availability of data from other countries, quality

10. **What are challenges to the use of cost data?**

11. **How important is cost as a factor in health policy decisions generally?**
    - **Probe:** Are there specific examples of where cost was an important in a decision?

*[**Interviewer guidance:** Please tell respondent the following questions may include some multiple choice questions]*

12. **Do you personally or your organisation use cost data for the following purposes *(more than one answer possible)*?**
    a. **Cost projections for strategic planning** or budgeting/ allocation across provinces or districts etc;
    b. **costing for cost-effectiveness analysis,** ie for resource allocation decisions, or adoption of a new technology;
    c. **expenditure reporting or efficiency analysis of service delivery,** ie, for benchmarking of expenditure or performance improvement, or budget execution tracking;
    d. **none of or in addition to the above, but rather for the following purpose *(interviewee to name)*:**

13. **How often do you think the results of economic evaluations or cost analyses are used in the South African health sector generally? (select one answer)**

Never	Rarely	Sometimes	All the time
1	2	3	4

Don't know	☐
n/a	☐

- **Probe:** Exactly how frequently, i.e. weekly, monthly, quarterly, annually etc.

Today's date					
d	d	m	m	y	y

Participant ID		

14. **How often do you think the results of economic evaluations or cost analyses are used in *your institution*? (select one answer)**

Never	Rarely	Sometimes	All the time
1	2	3	4

Don't know	☐
n/a	☐

- **Probe:** Exactly how frequently, i.e. weekly, monthly, quarterly, annually etc.

15. **How often do *you* use the results of economic evaluations or cost analyses in your programme decisions? (select one answer)**

Never	Rarely	Sometimes	All the time
1	2	3	4

Don't know	☐
n/a	☐

- **Probe:** Exactly how frequently, i.e. weekly, monthly, quarterly, annually etc.

16. **Does your organization need economic evaluation or cost data that you don't currently have?**
    - **Probe:** What type of data? for which types of interventions?

17. **Does your organization need any other data for programme planning that you don't currently have?**
    - **Probe:** What type of data? for which types of interventions?

18. **Amongst your organization's data needs, what are the nice to haves? What are the must haves?**

19. **Has your organization ever taken steps to improve the use of cost data? Please explain.**

20. **How often *would you like* to use data from economic evaluations or cost analyses in your programme decisions? (select one answer)**

Never	Rarely	Sometimes	All the time
1	2	3	4

Don't know	☐
n/a	☐

21. **What are currently the 3 main challenges in using data from economic evaluations or cost analyses in your program decisions?**

22. **Is your institution currently able to commission economic evaluations or cost analyses? (y/n)**

Today's date					
d	d	m	m	y	y

Participant ID		

Part C – Use of HE^{2}RO cost data

23. **We asked whether you had used outputs of economic analyses produced by HE^{2}RO staff above. Have you ever used any of these outputs to guide your organisation's programme decisions?**

24. **If yes, what types of decisions have been influenced by HE^{2}RO's economic evaluations?**

25. **Take me through the process of using HE^{2}RO data/outputs.**
    - **Probe:** Were you able to make timely decisions based on the cost data provided by HE^{2}RO?
    - **Probe:** Did the data come before or after you had to make the decision? If you received evidence post making a decision, were you able to adjust the policy/intervention based on the new evidence?
    - **Probe:** Was the data provided by HE^{2}RO useful?

26. **What are your thoughts on the format of the outputs?**
    - **Probe:** Was the format understandable? Please explain why or why not.

27. **What else would make this data more useful?**

28. **How was your interaction with HE^{2}RO staff during the project?**
    - **Probe:** How was the level of collaboration and feedback during the life of the project, if any?

29. **What did you like or dislike during your interaction with HE^{2}RO staff?**

30. **What did you think of the accuracy/quality of costing data supplied by HE^{2}RO?**
    - **Probe:** Were the cost data representative of real world costs? If not, where were the gaps?

31. **Please assess how much you agree with this statement: I am satisfied with the usefulness of economic evaluation and cost analysis results provided *by HE^{2}RO* in facilitating decision-making.**

Strongly Disagree	Disagree	Agree	Strongly Agree
1	2	3	4

Don't know	☐
n/a	☐

32. **Please assess how much you agree with this statement: I am satisfied with the usefulness of economic evaluation and cost analysis results provided *by other organisations* in facilitating decision-making.**

Strongly Disagree	Disagree	Agree	Strongly Agree
1	2	3	4

Don't know	☐
n/a	☐

Today's date					
d	d	m	m	y	y

Participant ID		

Part D – Future analyses

33. **What is your perspective on what type of cost data will be needed to make decisions under the National Health Insurance?**

34. **The NHI Bill states that under NHI there will be a government unit or agency responsible for Health Technology Assessments (HTA), a methodology closely related to economic evaluations. It also states that HTA will be used to inform the decision-making processes of the NHI Benefits Advisory Committee. This committee will then determine the benefits and types of services to be reimbursed by the NHI fund.**

What is your opinion on how academic institutions such as HE^{2}RO should contribute to the HTA process?

- **Probe:** What would the interaction with the HTA agency and/ or Benefits Advisory Committee look like?

35. **Should there be a cost-effectiveness threshold for inclusion of an intervention in the benefits package under NHI? If so, what should it be informed by?**
    - **Probe:** If yes, do you have a specific value in mind?
    - **Probe:** How else do you think different interventions and services should be prioritised?
    - **Probe:** What data would be needed for that?

36. **Is there anyone else you suggest we speak to?**

37. **Do you have any other questions for us or final comments?**

Many thanks for your time and insight!
