# Supplementary material for: Role of data from cost and other economic analyses in healthcare decision-making for HIV, TB and sexual/reproductive health programmes in South Africa
Source: Health Policy Plan. 2021 Jun 29;36(10):1545–51. doi: 10.1093/heapol/czab071 (PMC8597963; doi:10.1093/heapol/czab071)
Supplement: czab071_Supp [file czab071_supp.zip › Supplement 2_Quotes by theme.pdf]

# Quotes by Theme

| | |
|---|---|
| **Data Use** | |
| "… most of the time in the past it has been us just getting information coming through from them. We do the analysis here and then we use it and we move on without really getting them involved to get to, to empower them to be able to do much more on their own, on the at the lower level without our intervention." | a002 |
| "… So you need in house people who understand the importance of the cost data and how to use the cost data in their decision making. That has to happen from those who are employed by the Department of Health." | a015 |
| "When things are not costed then things are vague and then there is no accountability at either level." | a013 |
| **Challenges** | |
| Additional data inputs required | |
| "… you know the unit cost data is very useful but they actually need more information on how do we implement this. What are the HR needs? What are the cadre, staff we need, how many, what time frames?" | a004 |
| "One can never be sure because we are basing on estimates first of all and the other thing is these costs come mainly at national level. So then we are not able to replicate this for provincial level. So what then happens is the Treasury allocation they use a general formula that they use for allocating funds to the provinces. So you will find at that level where the burden is they don't get enough funding to actually deal with the burden and where there isn't that much of the burden they still get their normal allocation and the risk is that then they can then say there is money allocated and what can we use it for." | a022 |
| Data credibility | |
| "…most of the time the use of or acceptance of using the cost data is more of questioning the veracity or credibility of the unit costs that went into compiling that." | a002 |
| "And those processes are usually rigorous and in-, involve lots of stakeholder consultation and validation of inputs. And then in those circumstances the challenges are always gathering the inputs that we can trust, …" | a012 |
| Processes | |
| "… so you find that whatever decisions were made up there at the National level did not have much meaning for the district level, because of lack of communication, lack of coordination, but also limited involvement of the districts in those National decisions." | a005 |
| "… I think the brunt of the criticism that vaccine used to get, was from advocacy groups of saying we don't see us in this model, even though we're a critical enabler, they don't see us in this and if we're not in this, we don't give money and we don't give money then the gains made over time may start to slide." | a007 |

| | |
|---|---|
| "So a lot of government departments come up at a national level with policies and guidelines but they don't cost them and they just pass them down to the province and say here is this policy implement or they could be things on HIV and they say look, you have to do the following thirty things and you have a conditional grant and you got your own money so make it happen. So provinces are not supported in terms of how to make all of this happen from a set budget, how to prioritise within that and I always feel almost like national departments don't want people to know exactly how much it costs." | a013 |
| "I don't want to say the structures don't exist because there are opportunities for sort of ministries to get together and operationalise certain plans but the process is slow and cumbersome and the allocation of resources is therefore complicated because budgets are held within ministries. So you get almost a silo affect and then it is the competition for those resources and then the decision in terms of how to allocate those resources." | a017 |
| "I think you need to identify key stakeholders, including civil society more broadly from the inception of the exercise so that people know what it is that you are doing and they are more ready and willing to accept the results once the project is done. Forcing something down someone's throat is far more difficult if they didn't know from the get go that it is something that is needed and useful." | a017 |
| "District is more of a top down approach and it is not informed by the burden of disease that they need to deal with at district level." | a022 |
| Data literacy | |
| "People are not economically literate. So you have folks who use the data knowledgeably and then you have lots of folks who use the data poorly and that is a huge challenge, but fortunately in South Africa some of the key decision makers like the Treasury folks who make a lot of these decisions are super sophisticated. Once it rolls down to a decision that might be made by a provincial official or even a facility manager they are exactly the opposite. They don't have that level of experience and they may be given an economic analysis but not really understand how to use it and that is a big challenge." | a011 |
| "… one of the challenges with any cost data is the complexity with which it is presented. And, and as you can have these really fancy graphs and things like that and, and, and have these costs curves, but if nobody understands it –…" | a008 |
| Timeliness | |
| "… having the data that answers the exact question that you need to answer rapidly when answer rapidly when you need it. And that's always a challenge because the balance is with doing something really rigorous and rigour takes time, and so often times by the time the question is answered we've already moved on to, you know, step forth…" | a012 |
| "The challenge we have is that when we have questions we need answers like then but from you lot you want to do many, many months and sometimes years of research before we can get an answer." | a021 |
| Context (for data use) | |
| "The negatives, I think the challenges like what do we need for our research context and what do we need like right now –…" | a012 |
| "I think the biggest challenge for me is the gaps identified in the NSP and that is that we need to drive innovative financing methodology and that we need to drive evaluation cost effective analyses to drive efficiencies and evaluate efficiencies in government departments. I found that no government department wanted to be involved in those kind of evaluations." | a013 |

| | |
|---|---|
| "It kind of every time the message goes out there needs to be four or five bullet points below and this is assuming people will take it for a year and this is assuming, otherwise it does get taken out of context …" | a016 |
| **Enablers** | |
| Leadership | |
| "I think the most important element is that you have some leadership at the top." | a001 |
| Requirements | |
| "The data feeds into their principles and values in decision making and enables them then to make the choices." | a004 |
| "… so obviously costing is really important for a treasury …" | a006 |
| "… maybe more information and understanding on the unit cost and how that all is placed together." | a013 |
| "…there must be more and I think should be more proactive about what other people really need." | a013 |
| "… so actually seeing what the inputs are super important. I think some of the critical inputs like what adherence did one assume and what were the assumptions that went in based on what and if we use different assumptions, what would come out." | a016 |
| Support decision-making | |
| "… we need to know costs if when we, we're asking for example, to bring a new intervention." | a015 |
| "…I think it does certainly give decision makers a much higher level of confidence, that what they are then advocating and out their heads on the block for is going to work." | a016 |
| "… it is very important to focus on the individuals who are working for the Department of Health to understand how to use the cost data in the first place because we can't produce the cost data as well as make sure they are used in the appropriate manner. So it is really educating the health care officials or those decision makers on the best use of evidence in terms of costs and cost effectiveness …" | a018 |
